# The In-Plane Anisotropy of WTe_2_ Investigated by Angle-Dependent and Polarized Raman Spectroscopy

**DOI:** 10.1038/srep29254

**Published:** 2016-07-11

**Authors:** Qingjun Song, Xingchen Pan, Haifeng Wang, Kun Zhang, Qinghai Tan, Pan Li, Yi Wan, Yilun Wang, Xiaolong Xu, Miaoling Lin, Xiangang Wan, Fengqi Song, Lun Dai

**Affiliations:** 1 State Key Lab for Mesoscopic Physics and School of Physics, Peking University, Beijing, 100871, China; 2 Collaborative Innovation Center of Quantum Matter, Beijing, 100871, China; 3 National Laboratory of Solid State Microstructures, College of Physics, Nanjing University, Nanjing, 210093, China; 4 Collaborative Innovation Center of Advanced Microstructures, Nanjing University, Nanjing, 210093, China; 5 State Key Laboratory of Superlattices and Microstructures, Institute of Semiconductors, Chinese Academy of Sciences, Beijing, 100083, China; 6 MOE Key Laboratory for Nonequilibrium Synthesis and Modulation of Condensed Matter, Xi’an Jiaotong University, Xi’an, 710049, P. R. China

## Abstract

Tungsten ditelluride (WTe_2_) is a semi-metallic layered transition metal dichalcogenide with a stable distorted 1T phase. The reduced symmetry of this system leads to in-plane anisotropy in various materials properties. We have systemically studied the in-plane anisotropy of Raman modes in few-layer and bulk WTe_2_ by angle-dependent and polarized Raman spectroscopy (ADPRS). Ten Raman modes are clearly resolved. Their intensities show periodic variation with sample rotating. We identify the symmetries of the detected modes by quantitatively analyzing the ADPRS results based on the symmetry selection rules. Material absorption effect on the phonon modes with high vibration frequencies is investigated by considering complex Raman tensor elements. We also provide a rapid and nondestructive method to identify the crystallographic orientation of WTe_2_. The crystallographic orientation is further confirmed by the quantitative atomic-resolution force image. Finally, we find that the atomic vibrational tendency and complexity of detected modes are also reflected in the shrinkage degree defined based on ADPRS, which is confirmed by corresponding density functional calculation. Our work provides a deep understanding of the interaction between WTe_2_ and light, which will benefit in future studies about the anisotropic physical properties of WTe_2_ and other in-plane anisotropic materials.

Transition metal dichalcogenides (TMDs) have attracted a lot of attention because of their emerging and remarkable electrical, optical and mechanical properties^1^,^2^. In contrast to the widely studied TMDs like MoS_2_, WS_2_, MoSe_2_, WSe_2_ and MoTe_2_^3^,^4^,^5^, which are in-plane isotropous, WTe_2_ crystallizes in a distorted structure with an octahedral coordination around the metal, referred to as Td-polytype^6^. The recent discovery of non-saturating giant positive magnetoresistance and excellent thermoelectric behaviors in WTe_2_ arouses great interest in this material^7^,^8^,^9^. In addition, WTe_2_ is predicted to be a novel type of topological semimetal for further quantum transport studies^10^. The Td-WTe_2_ is constituted with triple-layer covalently bonded Te-W-Te atomic planes stacking along the *c*-axis through van der Waals interactions, as shown in Fig. 1a,b. The Td-WTe_2_ is strongly distorted from the ideal hexagonal net, because the off-centering W atoms form the slightly buckled W-W zigzag chains along the *a*-axis of the orthorhombic unit cell, as shown in Fig. 1c. The reduced crystal symmetry of WTe_2_ leads to the strong in-plane anisotropy.

Raman spectroscopy of both few-layer and bulk WTe_2_ was reported recently, which was crucial first step for WTe_2_ crystal structure characterization^6^,^11^,^12^,^13^,^14^. However, in order to further study the particular property of WTe_2_ and exploit related novel electronic and optoelectronic devices, more detailed study about few-layer and bulk WTe_2_ Raman spectra is still needed. In this work, we investigate the detailed Raman responses of both few-layer and bulk WTe_2_ flakes using the high-resolution angle-dependent and polarized Raman spectroscopy (ADPRS). The detected Raman modes are much more compared with the previous literatures^6^,^11^,^12^,^13^. By combining the symmetry analysis of the ADPRS results and the first principle calculation, we can accurately identify the symmetries of the detected modes and obtain the relation between their symmetries and lattice vibrations. We also identify the crystalline orientation of the WTe_2_ flakes based on the “in-plane anisotropy”, which is a precise and non-destructive all-optical method. Our work provides a deep understanding of the interaction between WTe_2_ and light, which will benefit in future studies about the anisotropic optical, electrical, and mechanical properties of WTe_2_ and other in-plane anisotropic materials^15^,^16^,^17^,^18^,^19^,^20^,^21^,^22^,^23^,^24^,^25^,^26^,^27^,^28^,^29^,^30^,^31^,^32^,^33^,^34^,^35^.

## Results and Discussion

**Figure 2:**
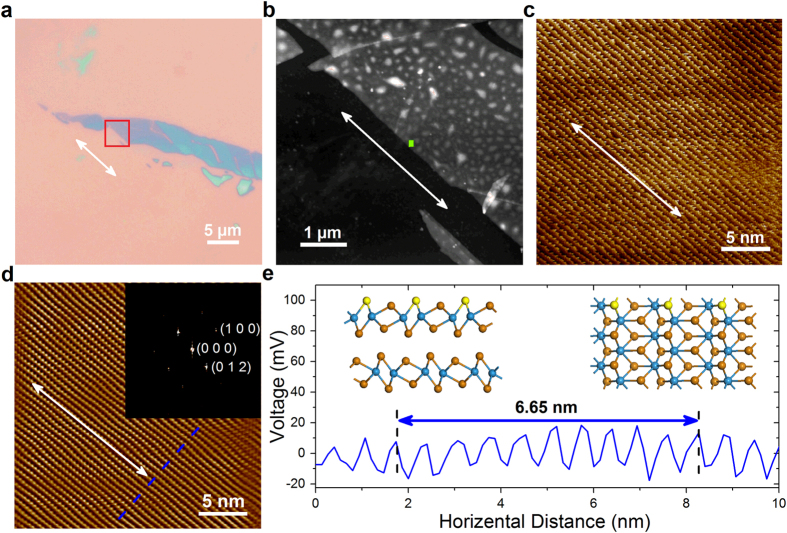
(**a**) Optical microscope image of the measured exfoliated WTe_2_ flake on SiO_2_/Si substrate. The white double-headed arrow indicates the well-defined edge, which is naturally formed after exfoliation. (**b**) AFM image of the red box area of the WTe_2_ depicted in (**a**). (**c**) HR-AFM image of the green box area of the WTe_2_ depicted in (**b**). (**d**) The smoothed HR-AFM image after FFT. The FFT image is shown in the inset. (**e**) The height variation profile perpendicular to the one dimensional chain along the dashed line in (**d**). The direction of well-defined edge in (**a**,**b**) and the direction along one dimensional chain in (**c**,**d**) are represented by white double arrows.

The Td-WTe_2_ bulk crystal used in this work was grown by the chemical vapor transport (CVT) method (more details in Method). The mono- and few-layer WTe_2_ were mechanically exfoliated on 300 nm SiO_2_/Si and quartz substrates (Supplementary Fig. S1) from the crystal. Figure 2a shows the optical microscope image of an as-exfoliated few-layer WTe_2_. Usually a well-defined edge (indicated by the white double-headed arrow) is naturally formed after exfoliation, due to the small cleave energy along the *a*-axis (*i*.*e*., the direction along the W-W chains). This is further confirmed by the quantitative atomic resolution force image probed by high-resolution atomic force microscopy (HR-AFM)^36^. Here, we define the *a*-axis as *x*-axis, the in plane direction perpendicular to it as *y*-axis, and the direction perpendicular to the 2D plane (*c*-axis) as *z*-axis. Figure 2b is an AFM image of the few-layer WTe_2_ (the red box area) in Fig. 2a. The corresponding HR-AFM image (the green box area) is shown in Fig. 2c. The smoothed HR-AFM image after the fast Fourier transform (FFT) is depicted in Fig. 2d. We can observe clearly one dimensional atomic chains parallel to the well-defined edge shown in Fig. 2a. The inset in Fig. 2d is the FFT image, where the distorted hexagon shape origins from the two different tungsten-tellurium bond lengths (2.7 Å and 2.8 Å). The height variation induced by the protruding tellurium atoms (highlighted in yellow in the inset) perpendicular to the one dimensional chains is shown in Fig. 2e. The average peak distance is about 6.65 Å, close to the lattice constant *b*.

**Figure 3:**
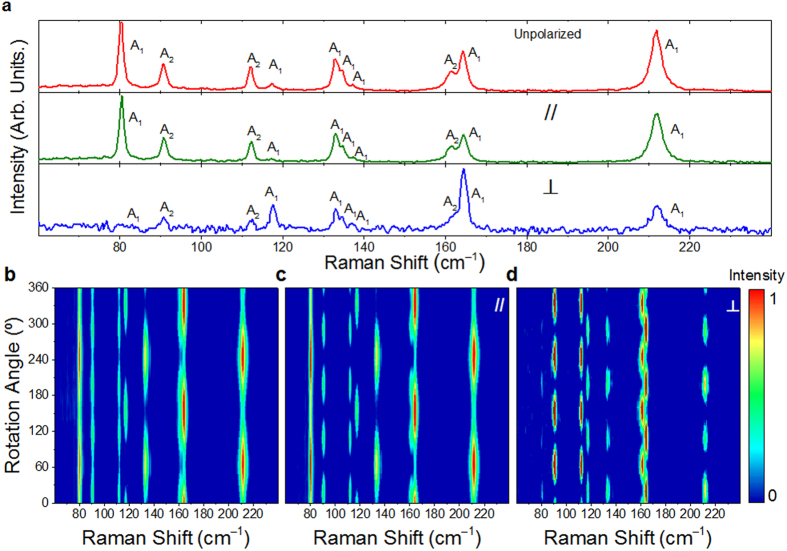
(**a**) Normalized Raman spectra measured at an angle where all modes appear in un-, parallel- and cross-polarized configurations. Angular dependence of the normalized Raman intensity spectra for the WTe_2_ flake measured in (**b**) un-polarized, (**c**) parallel-polarized and (**d**) cross-polarized configurations.

In the ADPRS measurement, a WTe_2_ flake on SiO_2_/Si substrate was initially placed with an arbitrary angle *θ*_*0*_ between the *x*-axis and horizontal direction. Herein, *θ*_*0*_ can be used to denote the crystalline orientation. We define *θ*_*0*_ to be positive (negative) value, when the *x*-axis is clockwise (anti-clockwise) compared to the horizontal direction (more details in Method and Supplementary Fig. S2). Figure 3a shows the Raman spectra of WTe_2_ in the un-, parallel- and cross-polarized configurations measured at an angle with the maximum number of Raman active modes. Altogether, ten Raman modes can be resolved. All of them can be well fitted by Lorentzian lineshape. Figure 3b–d show the angular dependences of the normalized Raman intensity spectra in the un-, parallel- and cross-polarized configurations, respectively. The sample rotation angle is in a range of 0–360°. The highest peak in each spectrum is used for normalization. We can see that, in the parallel-polarized configuration, the modes at ~80, 133, 135, 137 and 212 cm^−1^ yield 2-lobed shape with two maximum intensity angles at about 65° and 245°; the modes at ~117 and 164 cm^−1^ yield 2-lobed shape with two maximum intensity angles at about 155°and 335°; and the modes at ~91, 112 and 161 cm^−1^ yield 4-lobed shape with four maximum intensity angles at about 20°, 110°, 200° and 290°. In the cross-polarized configuration, all modes yield 4-lobed shape. The four maximum intensity angles for the modes at ~91, 112 and 161 cm^−1^ are *θ* = 65°, 155°, 245° and 335°, and those for the rest ones are 20°, 110°, 200° and 290°. In addition, we can see that the intensities of the three neighbored modes at 133, 135 and 137 cm^−1^ have similar angular dependent relations; while those of the two neighbored modes at 161 and 164 cm^−1^ have different angular dependent relations. The related angular dependent evolutions of these detected modes in the rotation angle range of 0–90° under the three polarized configurations are shown in Supplementary Figs S4–7, respectively. The ADPRS results for WTe_2_ with four representative different thicknesses (~3 nm, 10 nm, 25 nm and 40 nm) are shown in Supplementary Fig. S3. The results show that the anisotropic Raman spectra of WTe_2_ flakes have no clear thickness (≥3 nm) dependence.

**Figure 4:**
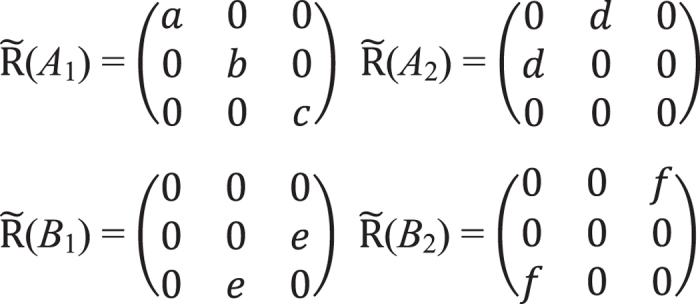
Raman tensor forms for all Raman active modes in bulk WTe_2_.

We can quantitatively analyze these observed anisotropic phenomena, based on the group theory, Raman tensors and density functional theory (DFT) calculations. According to symmetry analysis, the bulk Td-WTe_2_ belongs to the space group Pmn2_1_ and point group 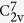
11,37. The unit cell of bulk Td-WTe_2_ contains two tungsten atoms and four tellurium atoms. There are 33 normal optical phonon modes at the Brillion zone center *Г* point, with irreducible representation as *Г*_bulk_  = 11*A*_1_ + 6*A*_2_ + 5*B*_1_ + 11*B*_2_, where all the vibration modes are Raman active. The 11*A*_1_, 5*B*_1_ and 11*B*_2_ modes are also infrared active. There exists a correlation between the Raman tensors of bulk and few-layer WTe_2_ (more details in Supplementary Information). For simplicity, we use the Raman tensors of bulk WTe_2_ (Fig. 4) to do the analysis^11^,^12^.

According to the classical Placzek approximation, the Raman intensity of a phonon mode can be written as^38^:


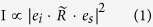


where *e*_*i*_ and *e*_*s*_ are the electric polarization unitary vectors of the incident and scattered lights, respectively, and 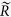 is the Raman tensor. The Raman tensors for all Raman active modes in bulk WTe_2_ are given in Fig. 4. Based on the Cartesian coordinates denoted above, the *e*_*i*_ and *e*_*s*_ are fixed in *xy* plane. For a sample with rotation angle of *θ* (clockwise rotation, as shown in Fig. S2), *e*_*i*_ = (cos(*θ* + *θ*_0_) sin(*θ* + *θ*_0_) 0) for the incident light, and *e*_*s*_ = (cos(*θ* + *θ*_0_) sin(*θ* + *θ*_0_) 0) and (−sin(*θ* + *θ*_0_) cos(*θ* + *θ*_0_) 0) for the scattered light in the parallel- and cross-polarized configurations, respectively. A phonon mode can only be detected when 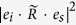 has non-zero value. Therefore, in the backscattering geometry, only *A*_1_ and *A*_2_ Raman modes can be observed. Using the above defined unitary vectors *e*_*i*_ and *e*_*s*_, as well as the Raman tensors of *A*_1_ and *A*_2_ modes, we can obtain the angular dependent intensity expressions for the *A*_1_ and *A*_2_ modes to be:


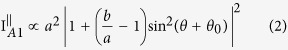



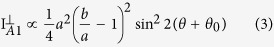



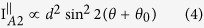



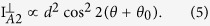


As the initial angle *θ*_0_ is fixed, the intensity of *A*_1_ or *A*_2_ mode is a function of the corresponding elements of Raman tensor (*a* and *b*) and the rotation angle *θ*. In the parallel-polarized configuration, the angular dependence for the intensity of *A*_1_ mode has two cases, both of which have a variation period of 180°. For *A*_1_ mode with *a *> *b*, the maximum intensity appears at *θ* = 180° − *θ*_0_ and 360° − *θ*_0_, corresponding to the incident light polarization parallel to the W-W chains. On the contrary, the minimum intensity appears at *θ* = 90° − *θ*_0_ and 270° − *θ*_0_, corresponding to the incident light polarization perpendicular to the W-W chains. For the *A*_1_ mode with *a* < *b*, the maximum intensity angles are *θ* = 90° − *θ*_0_ and 270° − *θ*_0_, and the minimum intensity angles are *θ* = 180° − *θ*_0_ and 360° − *θ*_0_, corresponding to the incident light polarization perpendicular and parallel to the W-W chains, respectively. In the parallel-polarized configuration, the angular dependence for the intensity of *A*_2_ mode has a variation period of 90° with the maximum intensity at *θ* = 45° − *θ*_0_, 135° − *θ*_0_, 225° − *θ*_0_ and 315° − *θ*_0_, and the minimum intensity at *θ* = 90° − *θ*_0_, 180° − *θ*_0_, 270° − *θ*_0_ and 360° − *θ*_0_. In the cross-polarized configuration, both of *A*_1_ and *A*_2_ modes have a variation period of 90°. The intensity of *A*_1_ mode (*A*_2_ mode) reaches its maximum (minimum) at *θ* = 45° − *θ*_0_°, 135° − *θ*_0_, 225° − *θ*_0_ and 315° − *θ*_0_, and reaches its minimum (maximum) at *θ* = 90° − *θ*_0_, 180° − *θ*_0_, 270° − *θ*_0_ and 360° − *θ*_0_. In addition, the normalized Raman intensities of the ten detected modes (except for *A*_2_ modes) in un-polarized configuration exhibit similar angular dependences to those in parallel-polarized configuration, as shown in Supplementary Fig. S8. It is worth noting that, as sample rotates, the full width at half maximum (FWHM) of each detected mode keeps almost constant, as shown in Supplementary Fig. S9.

**Figure 5:**
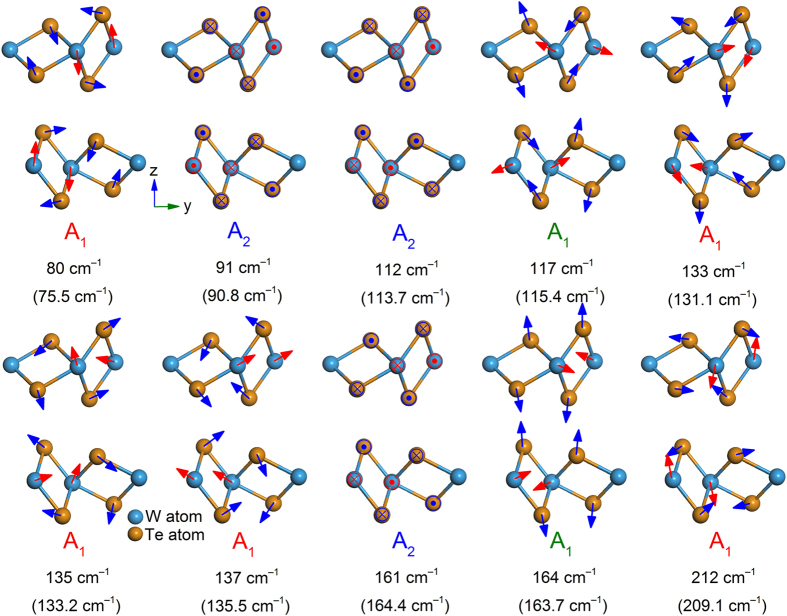
The calculated atomic displacements for the lattice vibrations of the ten detected modes in WTe_2_, together with their corresponding irreducible representations. The theoretical frequency is given below its experimental counterpart in each plot. The motions of W (Te) atoms are presented by red (blue) arrows.

According to the above analysis, we can use the ADPRS to identify the symmetries of the detected modes. The intensity variation periods for *A*_1_ modes are 180° and 90° in parallel- and cross-polarized configurations, respectively, while those for *A*_2_ phonon modes are 90° in both configurations. Therefore, seven phonon modes located at ~80, 117, 133, 135, 137, 164 and 212 cm^−1^ belong to *A*_1_ modes, and three modes located at ~91, 112 and 161 cm^−1^ belong to *A*_2_ modes. In addition, we find that when the incident polarization is parallel to the well-defined edge (*i*.*e*. parallel to the W-W chains) of the sample, the Raman modes at 117 and 164 cm^−1^ reach their maximum intensities. Therefore, we assign them to *A*_1_ modes with *a *> *b*. The rest *A*_1_ modes are with *a* < *b*. The lattice vibrations of all phonon modes are calculated by the density functional theory (DFT, more details in Method, Supplementary Fig. S11 and Table S1), and atomic displacements of detected ones are shown in Fig. 5. Because monolayer WTe_2_ (with space group *P21/m* and point group 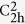) has different crystal symmetry with the bulk one, the 2-lobed modes in monolayer WTe_2_ can be labelled as *A*_g_, and the 4-lobed ones can be labelled as *B*_g_. Notably, there is no odd and even layer number dependence of crystal symmetry for WTe_2_. Therefore, for *N*-layer WTe_2_ (*N* ≥ 2, with space group *Pm* and point group 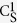), the 2-lobed and 4-lobed modes can be labelled as *A*′ and *A*″, respectively.

**Figure 6:**
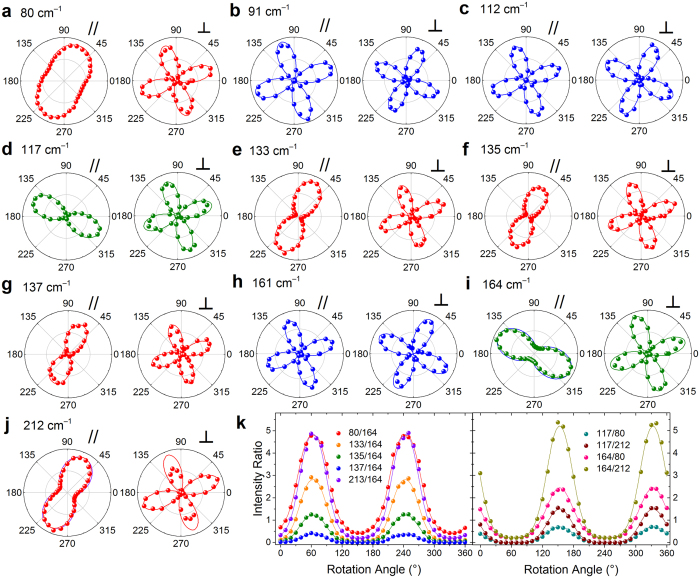
Angular dependence of the Raman intensities for the detected ten modes (**a**–**j**) in the parallel and cross polarization configurations. The experimental data are the scattered dots, and the curve fitting results are the solid lines. The intensity for each mode is normalized to its maximum value. Three types of modes, which have different angular dependent relations are colored in red, green and blue, respectively. (**k**) Angular dependence of the Raman intensity ratio for *A*_1_ modes with *a* < *b* and *a *> *b* in the parallel-polarized configuration. The curving fitting results are the solid lines.

Notably, according to above results, we can use the maximum intensity of the mode at ~164 cm^−1^ in un- and parallel-polarized configurations to identify the crystallographic orientation (*i*.*e*. the direction of W-W chains) rapidly and nondestructively. This is important in case that the well-defined edge of a few-layer WTe_2_ cannot be easily identified by the optical microscopy. In our case, it is represented by *θ*_0_ ~ 25°. The angular dependence of the normalized Raman intensities for the ten detected modes in the parallel- and cross-polarized configurations are shown in the polar plots in Fig. 6a–j. Notably, since the opposite angular dependent relations for *A*_1_ modes with *a* < *b* and *a *> *b*, their intensity ratio shows a clearer 2-lobed characteristic with sample rotating, as shown in Fig. 6k. By curving fitting Fig. 6k, we can obtain a more accurate *θ*_0_ to be 27.5°. The angular dependences of the Raman intensity ratios between other *A*_1_ and *A*_2_ modes, which are also helpful for identifying the crystallographic orientation, are shown in Supplementary Fig. S10.

It is worth noting that, the polar plots of *A*_1_ modes with higher frequencies (164 and 212 cm^−1^) in Fig. 6i,j cannot be well fitted by equation (2) (the blue and purple lines are the corresponding fitting results). In order to explain this, we consider the light absorption effect on the Raman tensor elements^38^,^39^. In an absorptive material, the elements of the Raman tensor should be complex numbers, with real and imaginary parts. In this case, the tensor elements of *A*_1_ and *A*_2_ can be written as


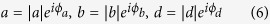


where *ϕ*_*a*_, *ϕ*_*b*_ and *ϕ*_*d*_ are the corresponding phases. Substituting in equation (1) with the unitary vectors *e*_*i*_ and *e*_*s*_ and the above Raman tensor elements, we can modify the angular dependent intensity expressions of the *A*_1_ and *A*_2_ modes as:


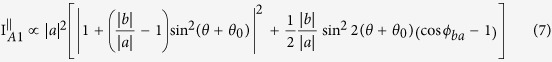



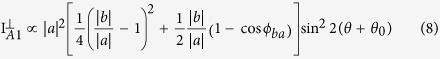



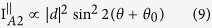



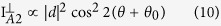


where *ϕ*_*ba*_ = *ϕ*_*b*_ − *ϕ*_*a*_ is the phase difference between the Raman tensor elements *b* and *a*. The expressions for *A*_2_ modes (equations 9 and 10) are identical to their counterparts (equations 4 and 5) obtained considering only real part of the Raman tensor elements. However, the expressions for *A*_1_ modes are different. We can see that the absorption effect on the ADPRS reflects in phase difference. The angular dependent intensities of *A*_1_ modes at 164 and 212 cm^−1^ can be well fitted by equations (7) and (8), as shown in Fig. 6i,j.

To further characterize the vibration direction of atoms for these detected modes. We choose defined *x*, *y* and *z* axes as the reference directions. Compared with the typical atomic displacements in 2H-type TMDs, such as MoS_2_, WS_2_, MoSe_2_ and WSe_2_ etc., the atomic displacements in WTe_2_ is relative complicated and disordered due to the lower symmetry. The related Raman tensor element ratios (*b*/*a*), phase differences, and shrinkage degrees for the ten detected modes are summarized in Table 1. Here, we define the shrinkage degree as the ratio of the maximum intensity and its orthogonal direction intensity in a polar plot. Considering the absorption effect, we can obtain the shrinkage degrees for *A*_1_ modes to be: 
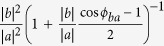
 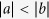 and 
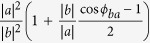
 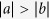, and those for *A*_2_ modes are closer to one. We find that the shrinkage degree reflects the vibrational tendency and complexity of detected modes. Specifically, the modes with smaller shrinkage degrees have the tendency to vibrate along the axes, and the ones with larger shrinkage degrees have the tendency to vibrate away from the axes. For three *A*_2_ modes, whose shrinkage degrees are close to one (minimum value), both W and Te atoms vibrate along the W-W chains (*x*-axis). In the case of *A*_1_ modes with relative small shrinkage degrees, for the *A*_1_ mode at ~80 cm^−1^, all the W atoms have the tendency to vibrate along the *z* axis, and half of the Te atoms have the tendency to vibrate along the *y* axis. For the *A*_1_ mode at ~164 cm^−1^, all the W (Te) atoms have the tendency to vibrate along the *y* (*z*) axis. For the *A*_1_ mode at ~212 cm^−1^, all the W (Te) atoms have the tendency to vibrate along the *z* (*y*) axis. For the rest four *A*_1_ modes with larger shrinkage degrees, their vibrations are more complicated. This finding may be applied for studying the complicated atomic vibrations in other anisotropic materials.

## Conclusion

In this work, we study the ADPRS of WTe_2_. Ten Raman modes are clearly resolved. Their intensities show periodic variation with sample rotating. We identify the symmetries of these detected modes by quantitatively analyzing the ADPRS results using the symmetry selection rules based on the Raman tensors, and do the curve fitting to the angular dependent intensities of them using the complex Raman tensor elements induced by absorption effect. We also provide a rapid and nondestructive method to identify the crystallographic orientation of WTe_2_. We find that the defined shrinkage degree based on ADPRS also reflects the vibrational tendency and complexity of the detected modes, which is confirmed by their atomic vibrations calculated by density functional theory. Our work provides a deep understanding of the interaction between WTe_2_ and light, which will benefit in future studies about the anisotropic optical, electrical, and mechanical properties of WTe_2_ as well as other in-plane anisotropic materials.

## Methods

### Growth of bulk WTe_2_

WTe_2_ single crystals were grown by the CVT method^8^. Stoichiometric W and Te powders were ground together and loaded into a quartz tube with a small amount of TeBr_4_ (transport agent). All weighing and mixing were carried out in a glove box. The tube was sealed under vacuum and placed in a two-zone furnace. The hot and cold zones were maintained at 800 °C and 700 °C, respectively, for 10 days. The crystal product appeared in cold zone.

### Measurements

The quantitative atomic resolution force image of WTe_2_ was measured by HR-AFM (Bruker Dimension Icon-PT). The angle- and polarization- resolved Raman spectra of exfoliated MoTe_2_ on 300 nm SiO_2_/Si substrate were measured by a commercial micro-Raman system (Horiba Jobin Yvon HR800) under the backscattering geometry. In order to obtain high-resolution spectra, we used a 100× object lens, and the grating with 1800 or 2400 grooves/mm. The exposure time is 100 seconds. The excitation wavelength was 633 nm, and the light power was below 400 μW. The incident light was polarized along the horizontal direction. The parallel- and cross-polarized configurations were constructed by placing an analyzer before the spectrometer.

### Density Functional Calculations

The calculations of phonon spectra were performed within local-density appreciation (LDA) using projector-augmented wave potentials. A 3 × 2 × 1 supercell was created and the interatomic forces were computed using the Vienna ab initio simulation package code with the small displacements method^40^. From these, force constant matrices and phonon frequencies were extracted using the PHONOPY Code^41^. The kinetic energy cutoff of the plane-wave basis was set to be 350 eV and 3 × 2 × 2 Monkhorst Pack grid was used in the phonon calculation.

## Additional Information

**How to cite this article**: Song, Q. *et al*. The In-Plane Anisotropy of WTe_2_ Investigated by Angle-Dependent and Polarized Raman Spectroscopy. *Sci. Rep.* **6**, 29254; doi: 10.1038/srep29254 (2016).

## Supplementary Material

Supplementary Information

## Figures and Tables

**Figure 1:**
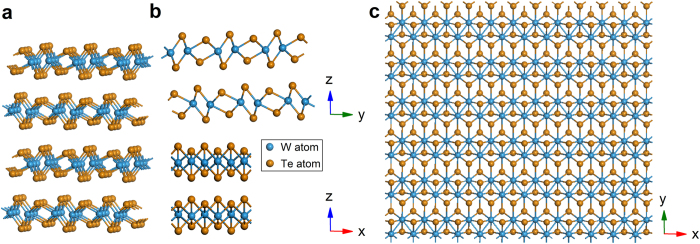
Crystalline structure of Td-WTe_2_, with (**a**) perspective view, (**b**) front view and side views, (**c**) top view.

**Table 1:** The irreducible representations, calculated frequencies Raman tensor elements ratio *b*/*a*, cos *ϕ*_ba_ and shrinkage degrees for all the detected phonon modes.

Experimental frequency (cm^−1^)	80	91	112	117	133	135	137	161	164	212
Irreducible representation	*A*_1_	*A*_2_	*A*_2_	*A*_1_	*A*_1_	*A*_1_	*A*_1_	*A*_2_	*A*_1_	*A*_1_
Calculated Frequency (cm^−1^)	75.5	90.8	113.7	115.4	131.1	133.2	135.5	164.4	163.7	209.1
Raman tensor elements ratio b/a	~0.71	–	–	~16.7	~0.30	~0.22	~0.10	–	~2.45	~0.49
cos *ϕ*_ba_	~1	–	–	~1	~1	~1	~1	–	~0.50	~0.68
Shrinkage degree	~1.98	~1.00	~1.00	~279	~11.1	~20.7	~100	~1.00	~3.72	~3.84
