# Supplementary material for: The In-Plane Anisotropy of WTe2 Investigated by Angle-Dependent and Polarized Raman Spectroscopy
Source: Sci Rep. 2016 Jul 11;6:29254. doi: 10.1038/srep29254 (PMC4941539; doi:10.1038/srep29254)
Supplement: Supplementary Information [file srep29254-s1.doc]

Supplementary Information for

**The In-Plane Anisotropy of WTe_2_ Investigated by Angle-Dependent and Polarized Raman Spectroscopy**

Qingjun Song, ^1,2^ Xingchen Pan, ^3,4^ Haifeng Wang, ^3,4^ Kun Zhang, ^1,2^ Qinghai Tan,^5^ Pan Li, ^6^ Yi Wan, ^1,2^ Yilun Wang, ^1,2^ Xiaolong Xu, ^1,2^ Miaoling Lin, ^5^ Xiangang Wan, ^3,4^ Fengqi Song, ^3,4^,*Lun Dai ^1,2^,*

^1^ State Key Lab for Mesoscopic Physics and School of Physics, Peking University, Beijing 100871, China.

^2^ Collaborative Innovation Center of Quantum Matter, Beijing 100871, China.

^3^ National Laboratory of Solid State Microstructures, College of Physics, Nanjing University, Nanjing 210093, China.

^4^ Collaborative Innovation Center of Advanced Microstructures, Nanjing University, Nanjing 210093, China.

^5^ State Key Laboratory of Superlattices and Microstructures, Institute of Semiconductors, Chinese Academy of Sciences, Beijing 100083, China.

^6^ MOE Key Laboratory for Nonequilibrium Synthesis and Modulation of Condensed Matter, Xi'an Jiaotong University, Xi'an, 710049, P. R. China.

Correspondence and requests for materials should be addressed to Fengqi Song & Lun Dai. (Email: [songfengqi@nju.edu.cn](mailto:songfengqi@nju.edu.cn), [lundai@pku.edu.cn](mailto:lundai@pku.edu.cn)).

**Raman tensors for *N*-layer (*N* is the number of layers) WTe_2_**

The odd and even layer number dependence of the symmetry is absent in *N*-layer WTe_2_ with stable Td phase, which is different from other TMDs with 2H phase. Monolayer (1-layer) WTe_2_ belongs to space group *P*21*/m* and point group C2 2h. With 6 atoms contained in its period unit cell, the irreducible representation of its phonon modes at the Brillion zone center *Г* point can be expressed as: *Г*_1-layer_ = 6*A*_g_ + 3*A*_u_ + 3*B*_g_ + 6*B*_u_. The bilayer and thicker few-layer WTe_2_ (*N*≥2) belongs to space group *Pm* and point group C1 S. With 6*N* atoms contained in its period unit cell, the irreducible representation of its phonon modes at *Г* point can be expressed as: *Г_N_*_-layer_ = 6*N A*′ + 12*N A*″. The bulk WTe_2_ belongs to the space group *Pmn*21 and point group C7 2v. With 12 atoms contained in its period unit cell, the irreducible representation of the phonon modes at *Г* point can be expressed as: *Г*_bulk_ = 12*A*_1_ + 7*A*_2_ + 6*B*_1_ + 11*B*_2_. Here, the *A*_g_, *B*_g_ and *A*_2_, are Raman active, *A*_u_ and *B*_u_ are infrared active, *A*_1_, *A*′, *A*″, *B*_1_ and *B*_2_ are both Raman and infrared active.

The Raman tensors
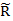
of the Raman active modes in 1-layer WTe_2_ can be expressed as:


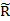
(*A*_g_) =
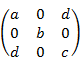


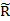
(*B*_g_) =
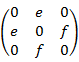


The Raman tensors
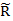
of the Raman active modes in *N*-layer WTe_2_ (*N*≥2) can be expressed as:


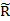
(*A*′) =
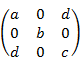


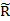
(*A*″) =
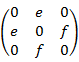


And the Raman tensors
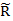
of the Raman active modes in bulk WTe_2_ (*N*≥2) can be expressed as:


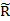
(*A*_1_) =
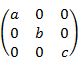

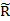
(*A*_2_) =
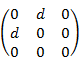


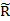
(*B*_1_) =
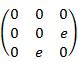

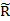
(*B*_2_) =
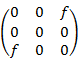


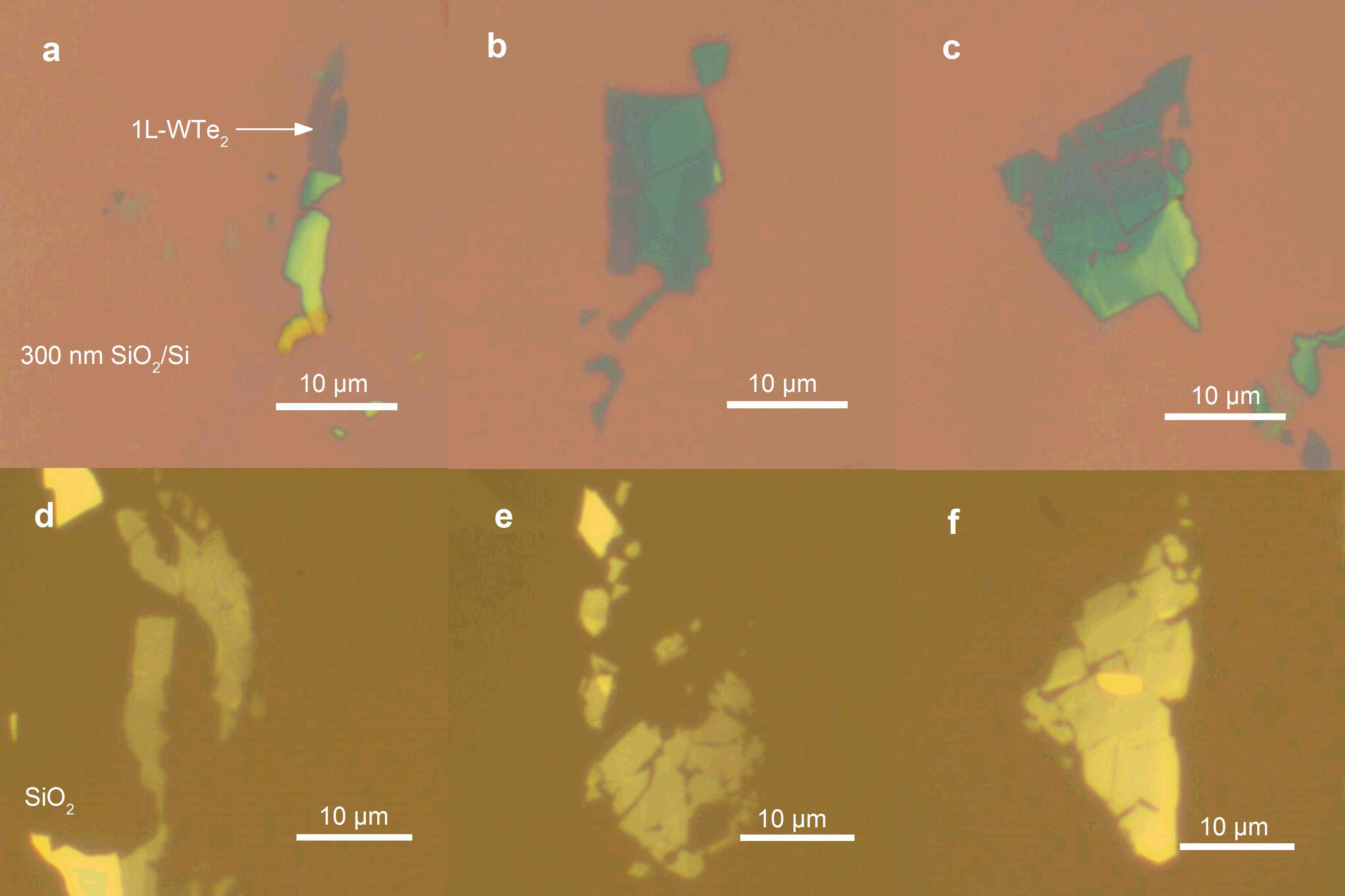


**Figure S1.** The optical microscope images of the monolayer and few-layer WTe_2_ on 300 nm SiO_2_/Si (a, b, c) and quartz substrates (d, e, f).


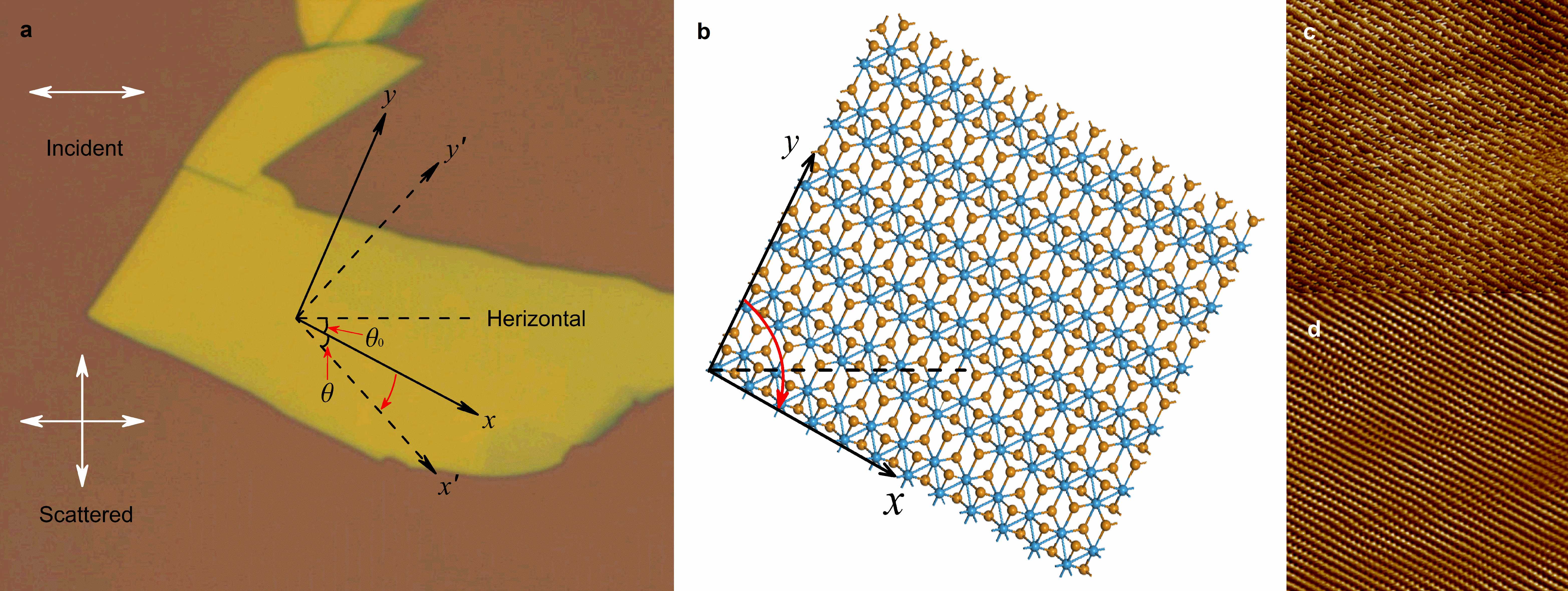


**Figure S2.** (a) Optical microscope image of the measured WTe_2_. The crystallographic axes, the initial angle between the incident light and the *x*-axis, the polarizations of the incident and scattered light are shown in the figure. (b) The top view of the distorted 1T crystalline structure of WTe_2_, whose axes are in accordance with those in optical microscope image. (c) The HR-AFM image and (d) the HR-AFM image after the FFT of the measured WTe_2_.


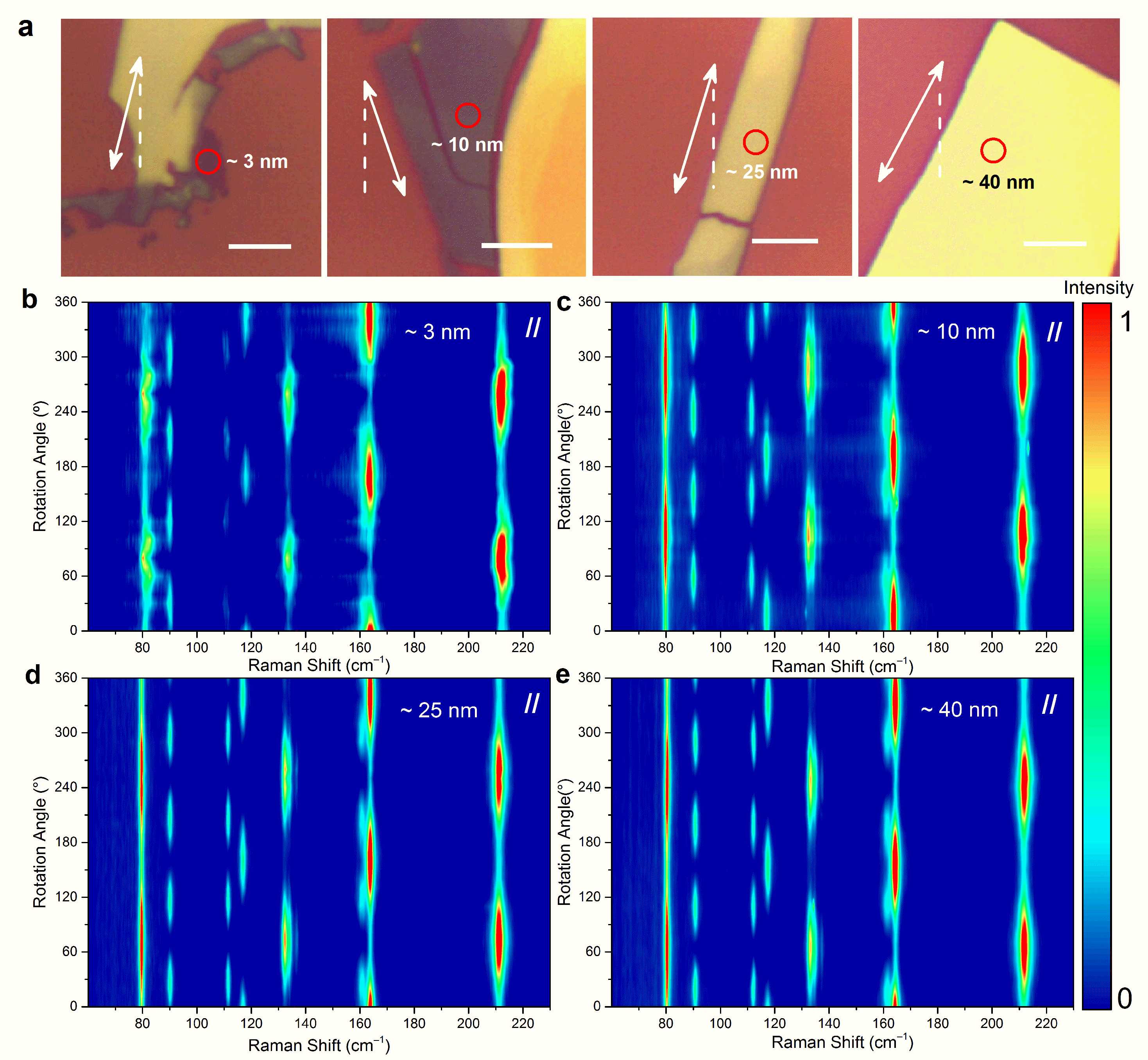


Figure S3-1. (a) Optical images of the thin WTe_2_ flakes with four different thicknesses. The red open circles label the sample measurement positions. The direction of the one dimensional W-W chains is labelled by white double arrows, and the incident polarization is vertical, as shown by the short dash line in each image. Scale bar: 5 μm. Angle dependence of the normalized Raman intensity spectra for the WTe_2_ flakes with the thickness of (b) ~3 nm, (c) ~10 nm, (d) ~25 nm, and (e) ~40 nm in the parallel-polarized configuration.

*
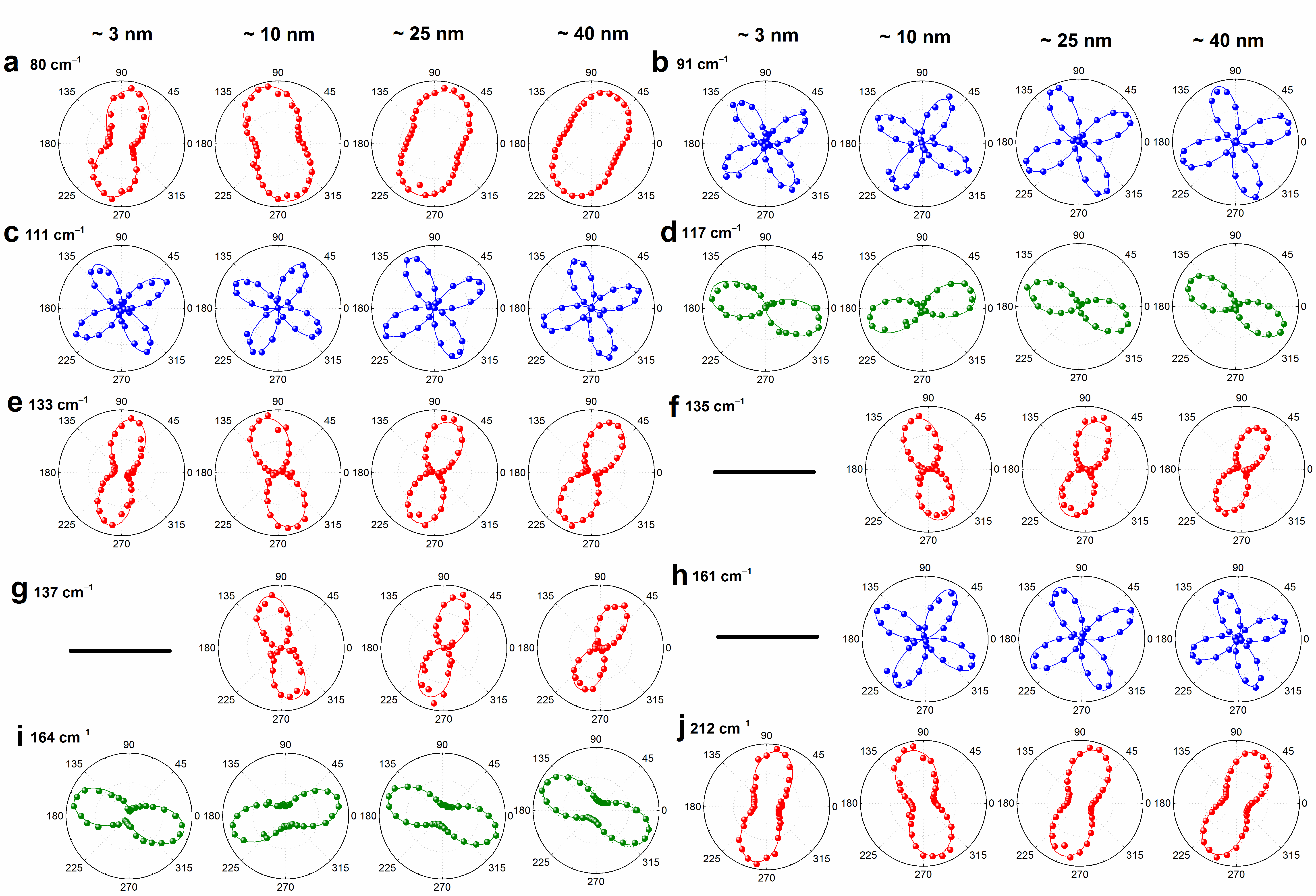
*

Figure S3-2. Angle dependence of the Raman intensities for the ten detected modes (a-j) in the four WTe_2_ flakes with different thicknesses (3nm, 10nm, 25nm, 40nm) in the parallel-polarized configuration. The scattered dots and the solid lines are the experimental data and the curve fitting results, respectively. The intensity for each mode is normalized to its maximum value. Three types of modes, which have different angular dependent relations are colored in red, green and blue, respectively. The modes at ~135, 137 and 161 cm^-1^ is too weak to their intensities.


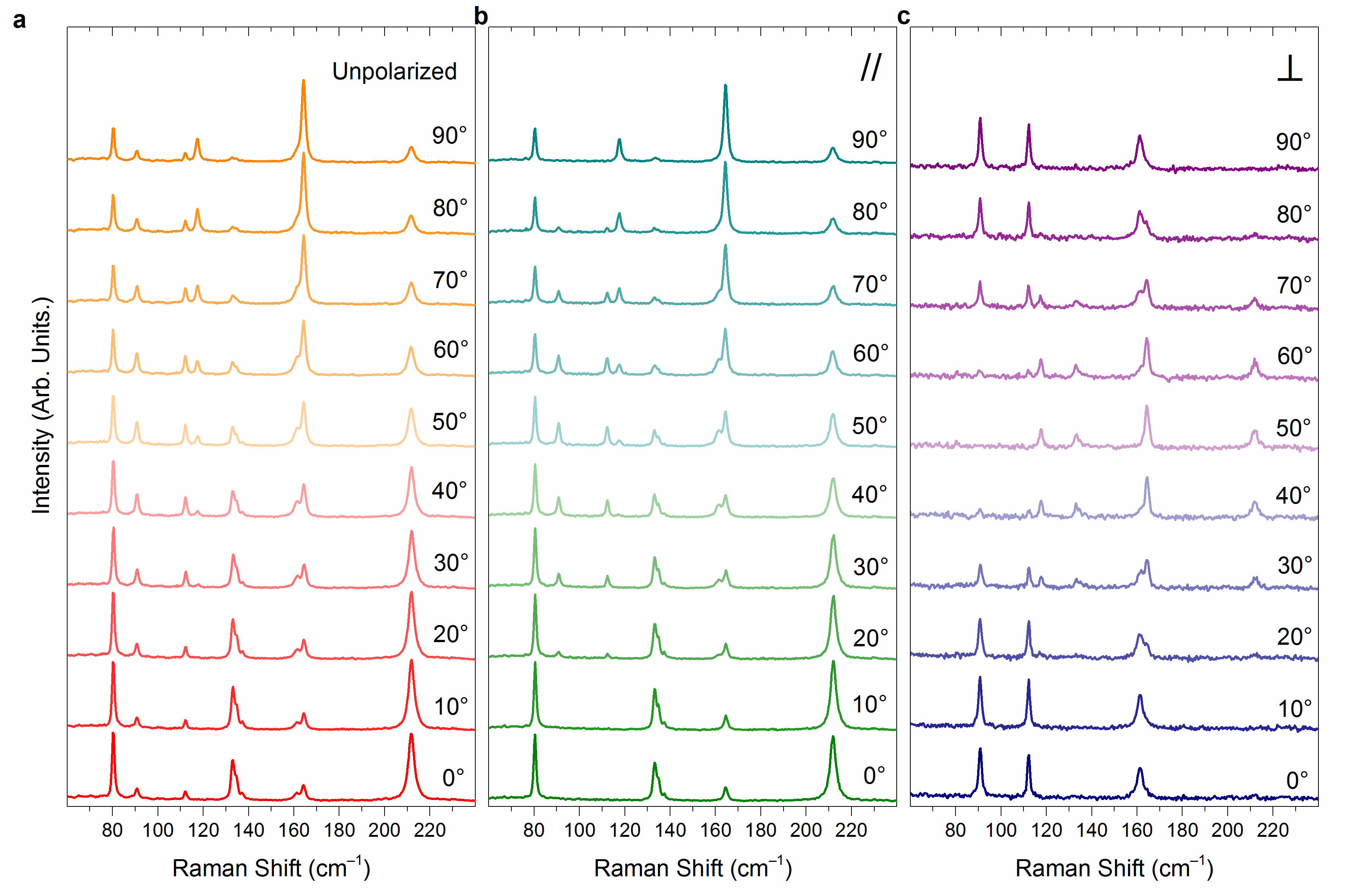


**Figure S4.** The related angular dependent evolutions of the Raman spectra in the rotation angle range of 0-90° under the un-, parallel-, and cross-polarized configurations.


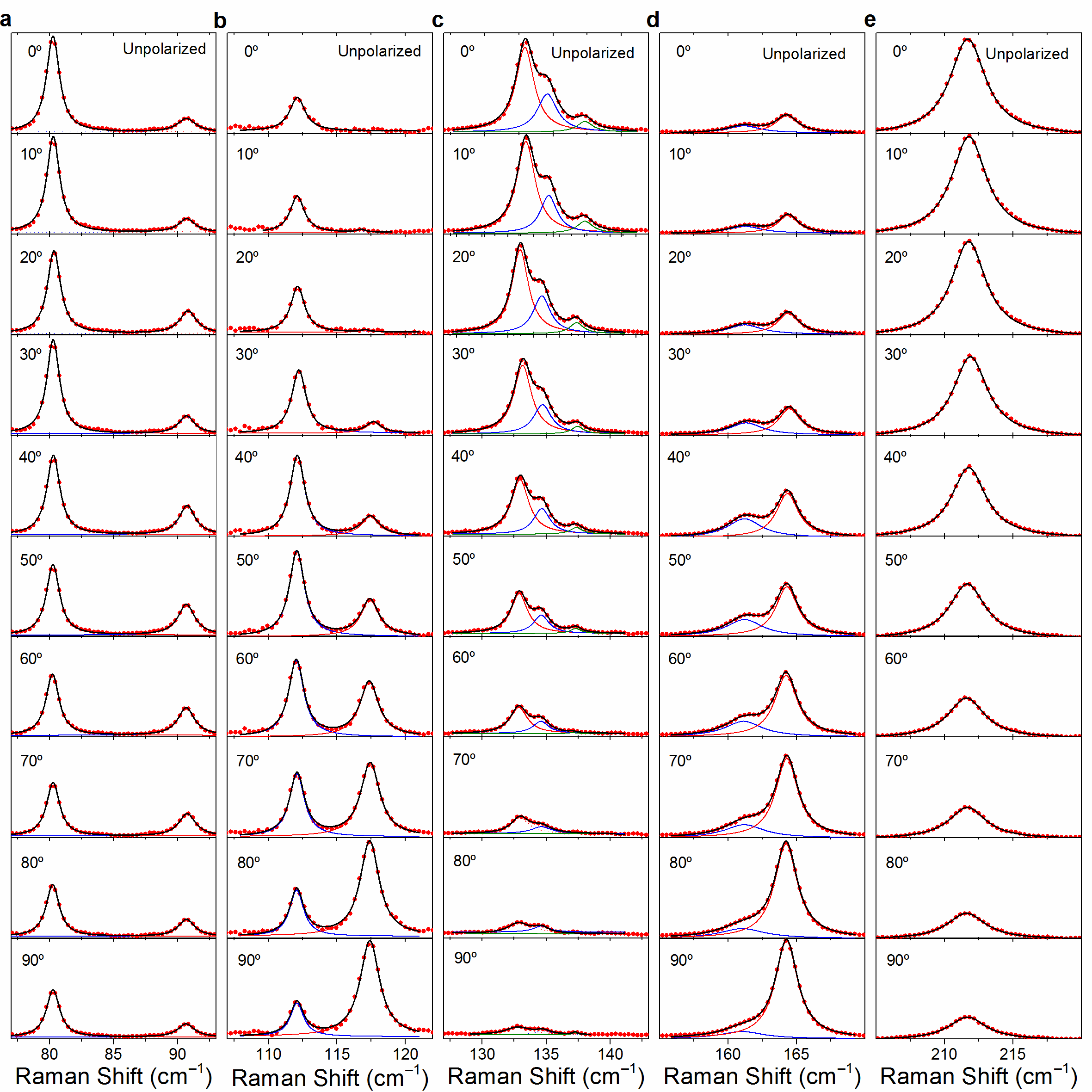


**Figure S5.** Normalized Raman spectra at different rotation angles (0-90°) in the frequency region of (a) 77-93 cm^−1^, (b) 107-123 cm^−1^, (c) 127-143 cm^−1^, (d) 155-170 cm^−1^ and (e) 207-223 cm^−1^ under the un-polarized configuration.


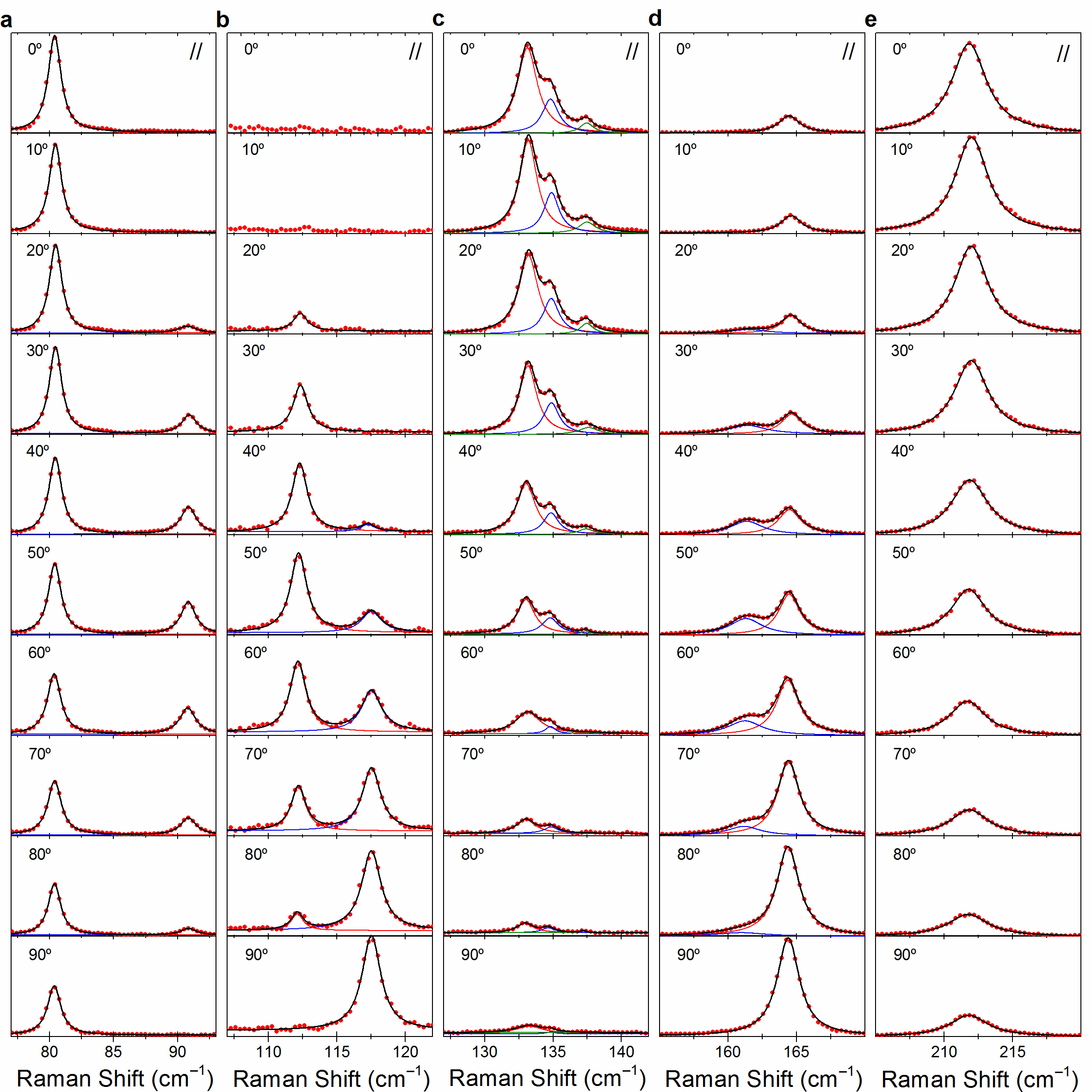


**Figure S6.** Normalized Raman spectra at different rotation angles (0-90°) in the frequency region of (a) 77-93 cm^−1^, (b) 107-123 cm^−1^, (c) 127-143 cm^−1^, (d) 155-170 cm^−1^ and (e) 207-223 cm^−1^ under the parallel-polarized configuration.


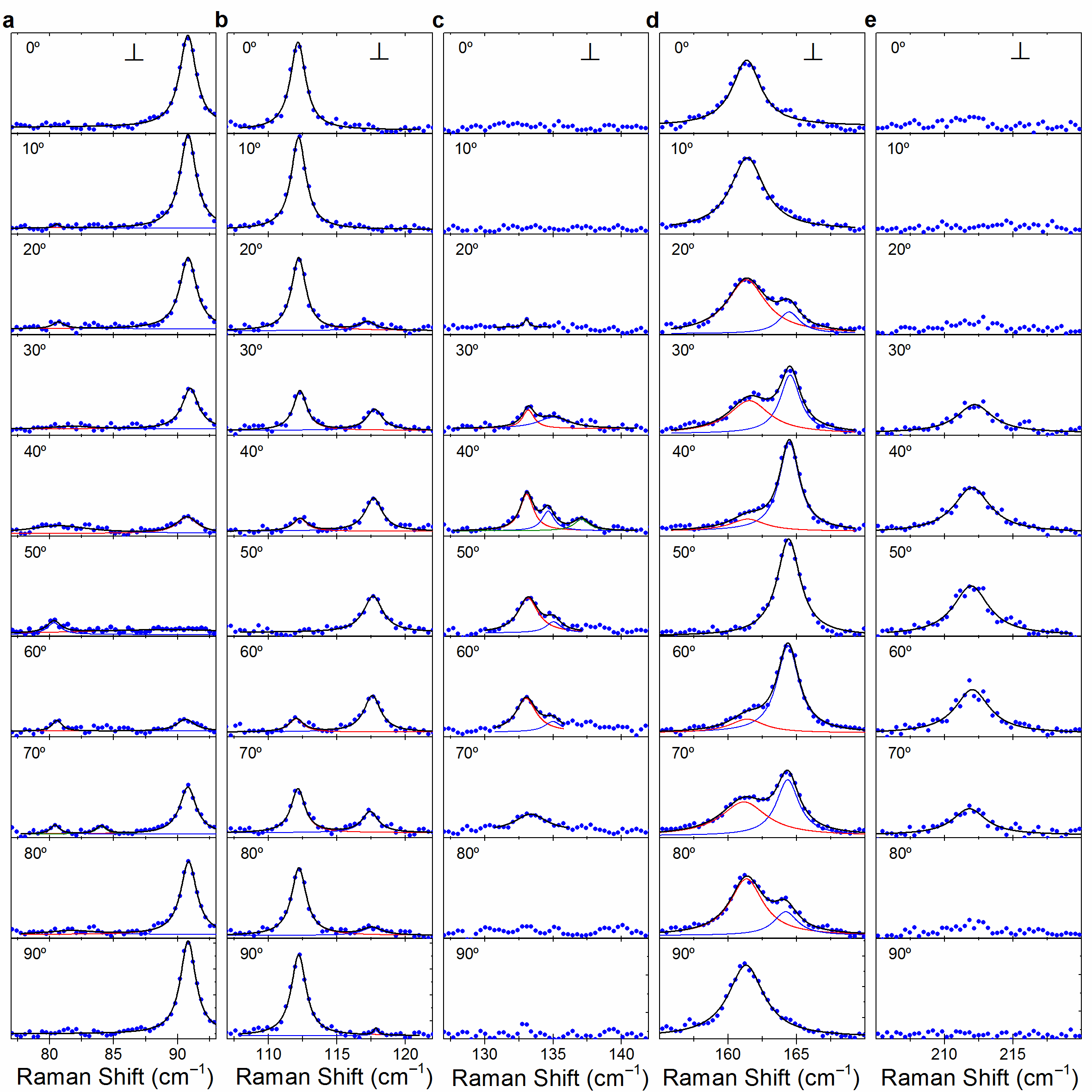


**Figure S7.** Normalized Raman spectra at different rotation angles (0-90°) in the frequency region of (a) 77-93 cm^−1^, (b) 107-123 cm^−1^, (c) 127-143 cm^−1^, (d) 155-170 cm^−1^ and (e) 207-223 cm^−1^ under the cross-polarized configuration.


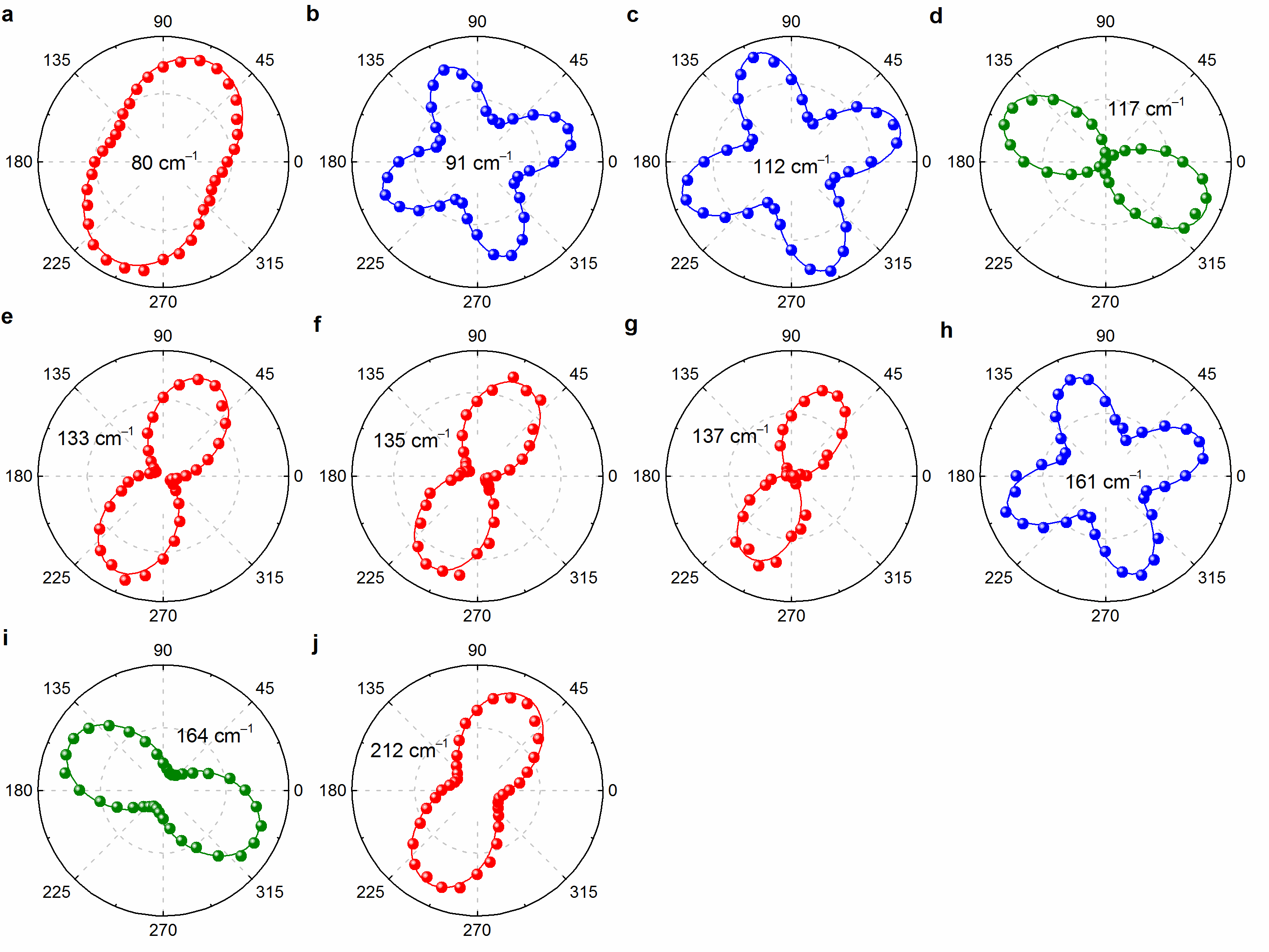


**Figure S8**. The polar plots for the angular dependence of the normalized Raman intensities for the ten detected modes in un-polarized configuration. The scattered dots are experimental data, and the solid lines are curve fitting results. The intensity for each mode is normalized to its maximum value. Three types of modes, which have different angular dependence relations are colored in red, green and blue, respectively.

The 2-lobed *A*_1_ modes in un-polarized configuration show similar angular dependence to those in the parallel polarization configuration, while the 4-lobed *A*_2_ modes in un-polarized configuration show significant different angular dependence with those in parallel polarization configuration. Their intensities can be well fitted by the equation


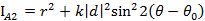


Comparing this with the equation (9) in the main text, we can see that an *r*^2^ is added in the above equation, which may origin from imperfect linear polarization of the laser.


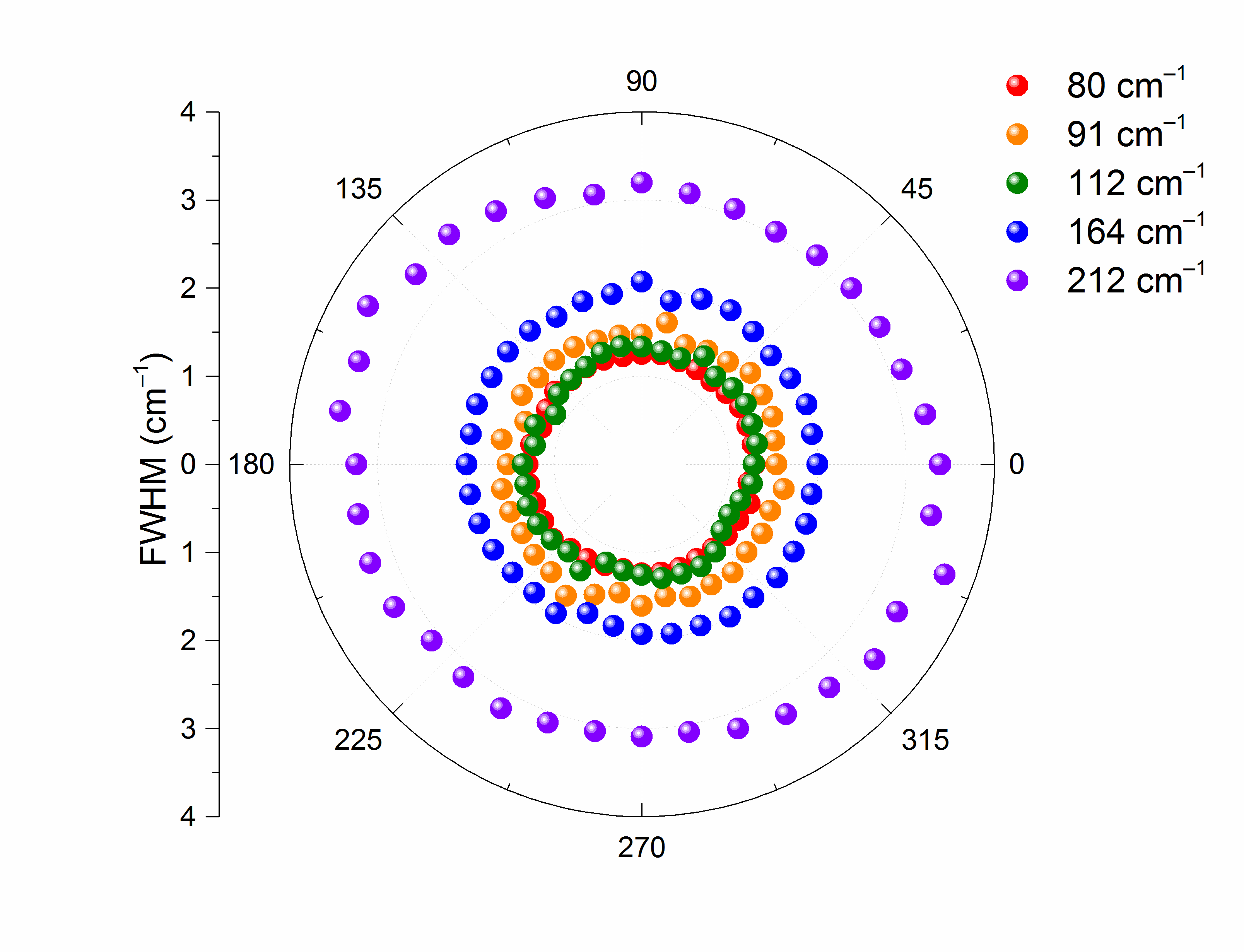


**Figure S9.** Angular dependence of the full width at half maximum (FWHM) for the modes located at 80 cm^−1^, 91 cm^−1^, 112 cm^−1^, 164 cm^−1^ and 212 cm^−1^ measured in the un-polarized configuration.

**
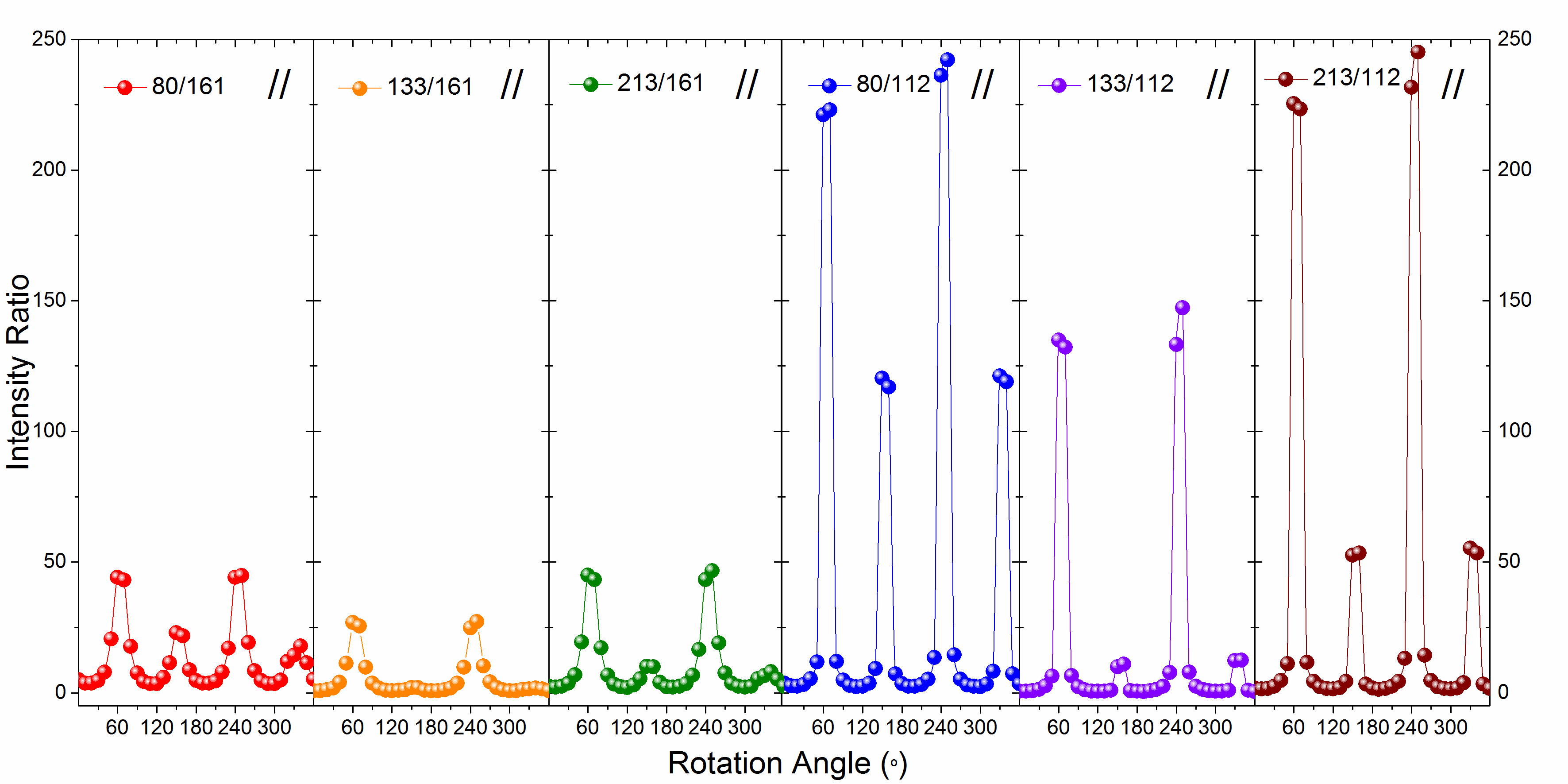
**

**Figure S10.** Angular dependences of Raman intensity ratios between some *A*_1_ and *A*_2_ modes in the parallel-polarized configuration.


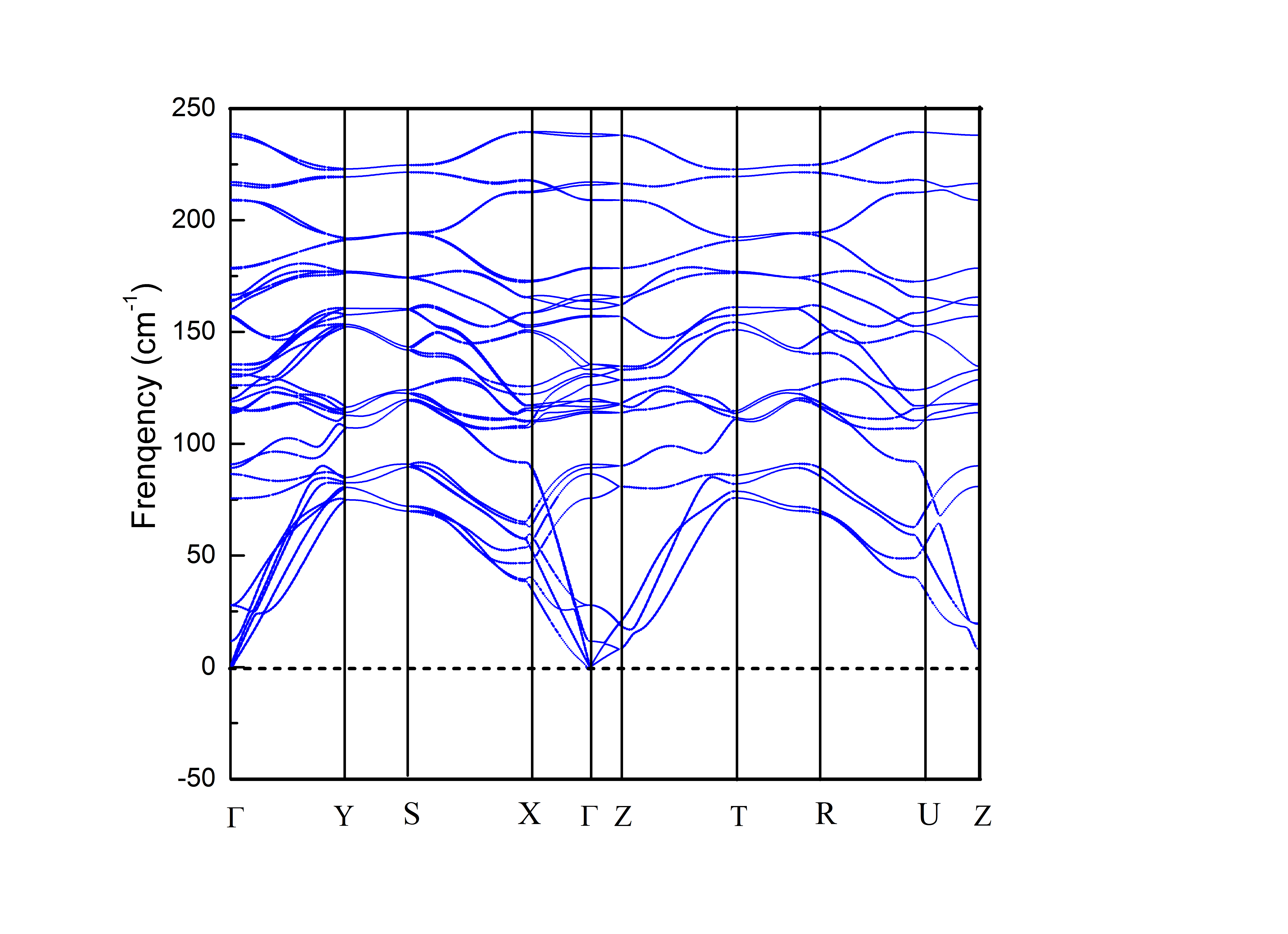


**Figure S11.** Calculated phonon dispersion curves along the Г-Y-X-Г-Z direction in the orthorhombic Brillouin zone.

**Table S1.** The irreducible representations, activity, calculated frequencies for 33 optical phonon modes in bulk WTe_2_ and the experimental frequencies for the ten detected modes.

| Irreducible representation | Activity | DFT Calculated frequencies  (cm^−1^) | Experimental frequencies  (cm^−1^) |
| --- | --- | --- | --- |
| *A*_1_ | R+IR | 9.2 |  |
| *A*_2_ | R | 27.6 |  |
| *B*_2_ | R+IR | 27.8 |  |
| *A*_1_ | R+IR | 75.5 | 80 |
| *B*_2_ | R+IR | 86.4 |  |
| *B*_1_ | R+IR | 89.2 |  |
| *A*_2_ | R | 90.8 | 91 |
| *A*_2_ | R | 113.7 | 112 |
| *B*_1_ | R+IR | 114.5 |  |
| *A*_1_ | R+IR | 115.4 | 117 |
| *A*_2_ | R | 116.5 |  |
| *B*_1_ | R+IR | 118.9 |  |
| *B*_2_ | R+IR | 120.1 |  |
| *B*_2_ | R+IR | 126.2 |  |
| *B*_2_ | R+IR | 130.0 |  |
| *A*_1_ | R+IR | 131.1 | 133 |
| *A*_1_ | R+IR | 133.2 | 135 |
| *B*_2_ | R+IR | 135.5 |  |
| *A*_1_ | R+IR | 135.5 | 137 |
| *A*_2_ | R | 156.7 |  |
| *B*_1_ | R+IR | 157.3 |  |
| *B*_2_ | R+IR | 160.1 |  |
| *A*_1_ | R+IR | 163.7 | 164 |
| *A*_2_ | R | 164.4 | 161 |
| *B*_1_ | R+IR | 166.6 |  |
| *A*_1_ | R+IR | 178.3 |  |
| *B*_2_ | R+IR | 178.7 |  |
| *B*_2_ | R+IR | 208.8 |  |
| *A*_1_ | R+IR | 209.1 | 212 |
| *A*_1_ | R+IR | 215.8 |  |
| *B*_2_ | R+IR | 217.0 |  |
| *A*_1_ | R+IR | 237.4 |  |
| *B*_2_ | R+IR | 238.6 |  |

**References**

1 Jiang, Y. C., Gao, J. & Wang, L. Raman fingerprint for semi-metal WTe_2_ evolving from bulk to monolayer. Scientific reports 6, 19624, doi:10.1038/srep19624 (2016).

2 Kim, Y. *et al.* Anomalous Raman scattering and lattice dynamics in mono- and few-layer WTe_2_. *Nanoscale* **8**, 2309-2316, doi:10.1039/c5nr06098b (2016).
